# Lack of Safe Drinking Water for Lake Chapala Basin Communities in Mexico Inhibits Progress toward Sustainable Development Goals 3 and 6

**DOI:** 10.3390/ijerph17228328

**Published:** 2020-11-11

**Authors:** Charlotte D. Smith, Kaitlyn Jackson, Hannah Peters, Susana Herrera Lima

**Affiliations:** 1 Division of Environmental Health Sciences, School of Public Health, University of California Berkeley, Berkeley, CA 94720, USA; kaitlynjackson@berkeley.edu (K.J.); hannah_peters@berkeley.edu (H.P.); 2 Departamento de Estudios Socioculturales, Instituto Tecnológico y de Estudios Superiores de Occidente, Tlaquepaque, Jalisco 45604, Mexico; shl@iteso.mx

**Keywords:** sustainable development goals, Lake Chapala, Mexico, CKD, diarrheal disease, water sanitation and hygiene, environmental health, environmental justice, Latin America, GIS

## Abstract

Background: Access to safe, affordable and accessible drinking water is a human right and foundational to the third and sixth World Health Organization’s Sustainable Development Goals (SDGs). Unsafe drinking water is a risk factor for chronic and enteric diseases. Both chronic kidney disease (CKD) and diarrheal disease are highly prevalent in the Lake Chapala basin, Jalisco, Mexico, suggesting disparities in factors leading to successful achievement of these two SDGs. Methods: This study aimed to assess progress towards SDG three and six in the Lake Chapala basin. Qualitative, quantitative, and geospatial data were collected between May and August of 2019 from three towns within the municipalities of Poncitlán and Chapala. Results: Ninety-nine households participated in this study. Water sampling analyses determined 81.18% of samples from water jugs (garrafones) and 70.05% of samples from tap water were contaminated with total coliform bacteria, often including *E. coli*. Additionally, 32% of garrafón samples and 61.9% of tap water samples had detectable levels of arsenic. Approximately 97.94% of respondents stated that they believe clean water is a human right, but 78.57% feel the Mexican government does not do enough to make this a reality. Conclusions: This mixed methods approach highlights water quality as a serious issue in communities around Lake Chapala, and demonstrates inadequate drinking water as a key hazard, potentially perpetuating the high disease burden of both CKD and enteric disease in the region.

## 1. Introduction

Globally, approximately three out of 10 people lack access to safely managed drinking water services and three billion lack access to basic sanitation services. Populations of low socioeconomic status have less access to improved water, sanitation, and hygiene (WASH) services, with rural settings disproportionately impacted [1,2]. Exposure to unsafe drinking water is one of the leading causes of water-borne enteric diseases [3]. Over the past decades both low- and middle-income countries have experienced an increase in the burden of non-communicable diseases (NCDs) [4]. Both communicable and non-communicable diseases can develop through repeated exposure to toxic or pathogenic agents in drinking water, highlighting that access to WASH and population health are deeply intertwined [5]. Safe, affordable and accessible WASH improves the health of people by improving their standard of living, educational outcomes, dignity and gender equality [2]. Access to safe, affordable and accessible drinking water is a human right, but barriers to access can create or further exacerbate health inequities. Both the United Nation’s 2000–2015 Millennium Development Goals (MDGs), and the subsequent 2015–2030 Sustainable Development Goals (SDGs) specifically target global improvements to WASH and population health [6,7].

The SDGs encourage the elimination of poverty through economic growth; address health, occupational and education needs; and aim to tackle climate change by 2030 [6,7]. Table 1 outlines SDG 3 and SDG 6, including the specified targets and indicator metrics which this study focused on to address the intersectionality between WASH and public health.

For prevention and treatment of many infectious and chronic diseases, access to safe WASH plays a critical role. The previous established indicator, defined by the MDGs to assess progress of WASH, was access to “improved” drinking water. Access to improved drinking water was defined as “access to sources which are protected against both fecal and environmental contamination” [9]. Although an important starting point, this definition is essentially a construction standard and did not inherently assure that a water source was “safe”. Therefore, SDG 6.1.1 addresses this fundamental issue by upgrading the metric for assessing the progress of WASH to safely managed drinking water. The term “safely managed drinking water” introduces three additional criteria into its definition; drinking water is (1) located within or around a home, (2) available when needed, and (3) free from fecal and chemical contamination [7].

Greater global awareness of public health issues related to water and sanitation is needed to achieve universal access to “safe” drinking water sources by 2030, as set by the sixth SDG [2]. Currently, the Joint Monitoring Program (JMP), created by the World Health Organization (WHO) and the United Nations Children’s Fund (UNICEF), monitors progress towards the WASH-related SDG targets through analyses of household surveys and national censuses [1,9,10]. However, in resource scarce settings where efficient data collection and analysis strategies are lacking in accurately assessing drinking water safety, new methodological approaches should be considered. We believe a mixed methods approach, including the collection and analysis of qualitative, quantitative, environmental and geospatial data, can characterize the disease burden and drinking water quality at the community level. Household surveys are often more feasible than individual level surveys in resource scarce settings by saving time, money and finite resources [9]. Additionally, by collecting data at the household level, hidden risk factors related to chronic and infectious disease that are inherent at the household or community level but not at the individual level are illuminated, which often includes water and sanitation.

### 1.1. Study Setting

The Lake Chapala basin is located in the states of Jalisco and Michoacán, Mexico. On the northern banks of Lake Chapala (in Jalisco), sociodemographic characteristics between towns varies widely. It is estimated that at least 20% of the population of the Lake Chapala basin has chronic kidney disease (CKD), nearly double the national estimates [11]. The mortality rate of diarrhea in the Lake Chapala basin is 39.5 per 100,000 population for children under five, three times the national mortality rate of children under five (10.8 per 100,000 population) [11,12].

Data for this study were collected from three towns along the Lake (San Pedro Itzicán, Mezcala, and Chapala) as a means of comparing across towns with varying socio-demographic profiles. Mezcala and San Pedro Itzicán are self-proclaimed indigenous communities in the municipality of Poncitlán. Each town has a total population of about 5000 inhabitants and are of a much lower socioeconomic status compared to other municipalities in the state of Jalisco [10]. Chapala is located 20 km from Mezcala, within the neighboring municipality of Chapala, and has approximately 20,000 inhabitants. Chapala is a tourist destination which leads to higher socio-economic status and is of interest to this study because it is more economically prosperous than most other towns in the Lake Chapala basin. Therefore, inhabitants are more likely to have the purchasing power to buy new bottles of water from major manufacturers as opposed to refilled bottles from local bottling companies. Furthermore, it gave us the opportunity to assess the socio-economic status in the interpretation of our findings.

In many regions where drinking water supply is intermittent and unreliable, households adapt by storing water in below ground cisterns or rooftop tanks rendering it highly vulnerable to microbial contamination [13]. This practice is very common in the Lake Chapala basin. Some households have water piped to their property from the town’s public water system, but the water service is not reliable due to daily shutoffs or complete loss of service. Properties without tap water receive trucked water deliveries, which fill rooftop tanks. These deliveries are also intermittent, arriving only a few days per week. Tap water in all three towns has been categorized as unsafe and citizens are advised against using it for drinking or cooking purposes. Therefore, households purchase 20 L water jugs (garrafones) as their primary drinking water source. In the Lake Chapala basin, many households purchase garrafones from local water purification companies costing between USD 0.75 and 1.00, or, if affordable, from name brands such as Ciel, Epura or Bonafonte, which cost as much as USD 2.00 per garrafón [14].

We assume that these major manufactures that sell water throughout Mexico, including the town of Chapala, are under at least some scrutiny from regulatory authorities. However, in Mezcala and San Pedro Itzicán (the poorer, less populated and more remote villages), we could not find any evidence of government oversight of the two local companies that refill recycled 20 L bottles with water from local wells. Communication with an employee of one of the companies confirmed that the bottles are not disinfected nor tested for microbial contamination before sale. To address this knowledge gap, we collected household water samples directly from the household’s primary and secondary drinking and cooking in-home water sources rather than from the local water purification companies. A similar technique was implemented in a study conducted in Belize in 2017, in which researchers asked household survey respondents for a glass of water that would be given to a child (“point of consumption”) and tested this water using a portable testing kit for *Escherichia coli* (*E. coli*) [9]. These measurements allowed researchers to identify specific areas for improvement in water supply at the household level.

### 1.2. Research Aims

We designed a study that utilized a mix of quantitative, qualitative, and geospatial methods, synthesizing the results from focus groups, survey data, water quality parameters and geospatial analysis to assess progress towards the third and sixth SDGs in the region of the Lake Chapala basin, Jalisco, Mexico. Using this mixed methods approach, we observed the availability of safely managed drinking water, household and community water use practice, and the household diarrhea and chronic disease burden. The study’s aims were to evaluate the effectiveness of this approach in assessing safely managed drinking water, disease prevalence, and overall progress towards the third and sixth SDGs in this region to ensure key monitoring SDG targets are met by 2030. We assessed the arsenic levels in primary and secondary water sources because they have been associated with CKD. *E. coli* a surrogate for fecal contamination has been associated with diarrheal disease. This study was a collaborative research endeavor between the University of California, Berkeley and El Instituto de Tecnológico y de Estudios Superiores de Occidente (ITESO) in Guadalajara, Jalisco, México.

## 2. Methods

### 2.1. Study Design and Sampling

**Figure 1:**
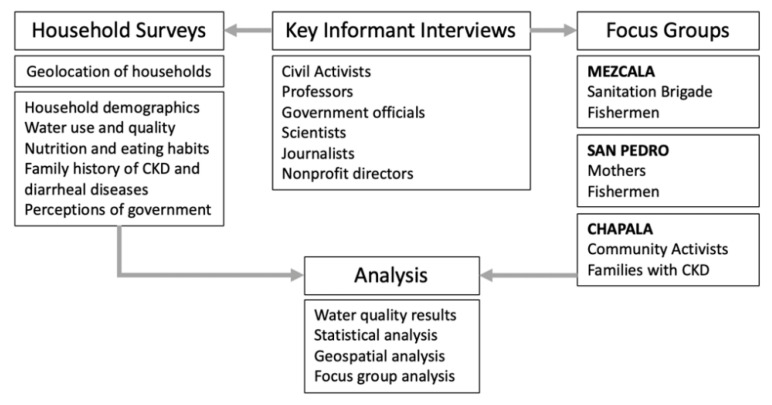
Mixed methods study design.

The study had five levels of data collection: (1) key informant interviews, (2) focus groups, (3) household surveys, (4) household water samples, and (5) household geospatial data collection (Figure 1). Information and samples were collected between May 2019 and August 2019 by University of California, Berkeley graduate students through collaboration with local community leaders and ITESO. The survey instrument and study protocol were approved by the ethics committee of the University of California, Berkeley Institutional Review Board (Protocol #2019-03-11986), which included all investigators from Berkeley and ITESO.

### 2.2. Household Survey Development

Between May and August 2019, a total of 13 key informant interviews and six focus groups were conducted. Key informant interviews with local researchers, government representatives, community organizers, activists, and journalists were used to inform the research team on the conditions of the communities, as well as adjust the survey instrument to be more culturally appropriate and context specific. Key informants were asked about their involvement in the three participating towns related to water, sanitation, hygiene, and health issues, perceptions of environmental factors contributing to high disease burden within these communities, and personal recommendations to reduce prevalence of CKD and enteric diseases.

Six focus groups were conducted with various groups of community members in the participating towns. The focus groups consisted of 8–12 individuals over the age of 18 who resided in one of the three towns. Participants were asked to discuss their perceptions surrounding water quality, community-level health, potential hazards that could negatively impact the health of these communities in general, and then specifically CKD and diarrheal diseases.

### 2.3. Household Surveys

To recruit households to enroll in our study, the survey team located themselves in each town center and participants were recruited if they chose to engage with a surveyor and met eligibility criteria (lived in and currently had a residence in San Pedro Itzicán, Mezcala, or Chapala, were 18 years of age or older and were ethnically Mexican). Participants provided written consent before beginning the interview. Household surveys were completed using the location-enabled survey platform, Survey123 (ESRI, Redlands, CA, USA).

The household survey, which included five sections, was built relying on ITESO’s local expertise as well as feedback from key informants and focus groups. Respondents answered all sections on behalf of their household members. Participants were made aware that their personal information would be de-identified prior to responding to these questions, to ensure they felt comfortable providing honest answers. Individual level demographic information was captured to assess age and sex of the respondent, and household members. Household size, income, employment, gender distribution, and age makeup of the other members was reported by the head of household to capture important demographic information of the population. Questions on water access and usage asked participants about their primary and secondary sources of water used for drinking, cooking, cleaning, and bathing purposes (purchased, bottled, piped, delivered or private well); brands of water available; perceptions of drinking water and health; water use practices; in-home water treatment practices. Household diet and food security were assessed to identify prevalence of food insecurity and/or malnutrition, which are common risk factors for chronic conditions such as CKD, hypertension and diabetes. Household history of disease was assessed through questions related to symptoms of CKD and diarrheal diseases suffered by each household member. The last section of this survey included five qualitative survey questions related to perceptions of government involvement within the community. These questions were developed through in-depth focus groups with local community members and leaders.

There was a total of 322 possible survey questions (including branched questions). Excel (Microsoft, Seattle, WA, USA), RStudio (RStudio, Inc., Boston, MA, USA), and ArcGIS Pro 2.5.2 (ESRI, Redlands, CA, USA) were used for data cleaning, statistical and geo-spatial analyses. Prevalence estimates for the number of households with at least one member with diarrheal symptoms within the last 2 weeks, and/or with at least one member diagnosed with CKD were calculated from the study sample. No hypothesis nor inference testing was conducted on household survey results. This household survey tool was the first of its kind implemented within these specific communities with the aim of producing descriptive statistics on community level water management and disease burden. The primary purpose of data collection was to document whether the drinking water in these communities (including the locally produced bottled water) is contaminated. For this reason, survey results are reported as counts and percentages.

### 2.4. Water Quality Sampling

Total coliform bacteria were measured to indicate efficiency of disinfection of source water and general microbial quality. *E. coli* was measured as it is the best indicator of fecal contamination [15]. World Health Organization (WHO) guidelines and the Mexican Standards for Drinking Water Quality require that total coliform bacteria and *E. coli* should not be detectible in any 100 mL drinking water sample [15,16].

Household water samples were taken directly from household’s primary and secondary drinking and cooking in-home water sources (as identified by the respondent). The samples were tested in the field for total chlorine and free chlorine, and at ITESO’s laboratory facilities for arsenic, using Hach^®^ product numbers 2,745,050 and 2,800,000 respectively (Hach, Loveland, CO, USA). The method detection limits for total and free chlorine are 0.05 mg/L and 10 μg/L for arsenic using these field kits. The presence of total coliform bacteria and *E. coli* was determined with Colilert^TM^ (IDEXX, Westbook, ME, USA). Only one sample per water source was obtained due to water scarcity within households. Samples were transported on ice and analyzed for total coliform bacteria and *E. coli*. Quality control and quality assurance (QA/QC) field samples consisting of duplicates and negative controls (field blanks) were obtained during each day of sampling by each sample collector. These samples were incubated and analyzed along with the test samples. Duplicate samples matched the test sample and field blanks consistently showed satisfactory negative findings (Quality Control Data available upon request).

Counts and percentages of water quality results of dichotomous variables were stratified by the source (20 L “garrafón”, tap, 55-gallon drum) and reported. “Present” results indicate a household that had at least one water sample from the specified source with the contaminant present. “Absent” results indicate a household did not have any water samples from the specified source with the contaminant present.

### 2.5. Geospatial Analysis

Households with positive *E. coli* results or the presence of arsenic were mapped using ArcGIS Pro 2.5.2 (ESRI, Redlands, CA, USA). The Getis Ord Gi* statistic was used to determine whether the households or the positive *E. coli* results were randomly distributed (or clustered) in any of the three towns. The statistic could not be applied to the arsenic data because the total number of positive arsenic samples was too low.

## 3. Results

### 3.1. Descriptive Statistics

A total of 99 households were recruited from Mezcala (N = 32), San Pedro Itzicán (N = 35), and Chapala (N = 32). Sociodemographic characteristics of the study sample are presented in counts (N) and percentages (%) in Table 2. Overall 16.16% of households had at least one member diagnosed with CKD (56.25% in Mezcala, 31.25% in Chapala and 12.5% in San Pedro Itzicán). Additionally, 31.31% of households reported at least one member with diarrhea symptoms within the last two weeks prior to participating in our household survey (26.37% in Mezcala, 38.24% in Chapala and 35.29% in San Pedro Itzicán)

Descriptive statistics outlining water use practices among participating households are shown in Table 3. The most common drinking and cooking water source among our study population was the purchased 20 L water jugs.

Qualitative survey questions centered around perceptions of government support for increased access to clean water in all three towns. Results suggest that survey respondents are dissatisfied with their water quality and with government efforts to ensure the access to safely managed drinking water. Almost all study participants believed safe water access is a human right.

### 3.2. Water Quality Analysis

To assess if household drinking water is safely managed drinking water, indicators for fecal and chemical contamination were measured. Ninety-eight percent of respondents reported that garrafones were their primary drinking water source (Table 3) and for this reason, garrafones are referred to herein as the household’s primary drinking waters source. Tap and 55-gallon water drums are referred to as household secondary water sources. The total number of samples with total coliform bacteria, *E. coli*. or arsenic present in samples taken from each household water source (garrafón, tap and 55-gallon drum). All water quality analyses for each source are listed in Supplementary Material.

Of the 85 total drinking water samples from garrafones analyzed for microbial contamination, 81.18% were contaminated with total coliform bacteria and 23.26% were contaminated with *E. coli*, exceeding Mexican standards. There was a difference in both total coliform bacteria and *E. coli*, in the municipality of Poncitlán in which Mezcala and San Pedro Itzicán are located versus the town of Chapala as follows: 89%, 94% and 58% of samples were positive for total coliform bacteria and 56%, 13% and 31% of the samples were positive for *E. coli* in the three towns, respectively. The higher numbers of positive total coliform bacteria samples in Mezcala and San Pedro Itzicán were most likely due to purchase of improperly treated garrafones from local vendors as opposed to name-brand bottled water, available in Chapala. The fact that bacterial contaminants were found in garrafones in all three towns, however, points to the need to examine the point or points at which contamination occurs (during initial filling—i.e., from the source, during refilling, or within the home after the bottle has been opened).

**Figure 2:**
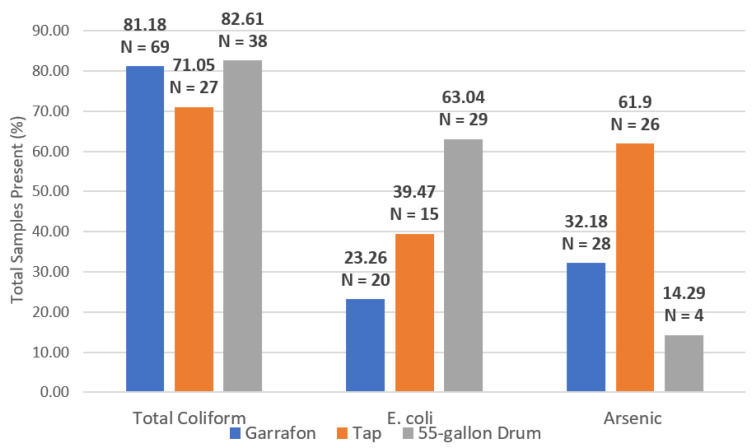
Percent (%) and number (N) of total samples with contaminant present for total coliform bacteria, *E. coli* and arsenic in 20 L garrafón, tap, and 55-gallon drum water samples.

Of the 38 tap water samples analyzed for microbial contamination, 70.05% were contaminated with total coliform bacteria and 39.47% were contaminated with *E. coli*. Only 17% of tap water samples showed any indication of a total chlorine residual. Of the 42 tap water samples analyzed for chemical contaminants, 61.9% had an arsenic concentration of 10 μg/L (the Method Detection Limit of the test). Of those samples with detectible arsenic, 47% had arsenic concentrations greater than 10 μg/L (Figure 2). Lastly, of the forty-six 55-gallon water drum samples analyzed for microbial contamination, 82.61% were contaminated with total coliform bacteria and 63.04% were contaminated with *E. coli*. A multivariate analysis of arsenic and *E. coli* along with potential confounding exposure variables, including diet and occupation, did not indicate an association between those variables and either CKD or frequency of diarrheal disease *p* > 0.05.

### 3.3. Geospatial Analysis

**Figure 3:**
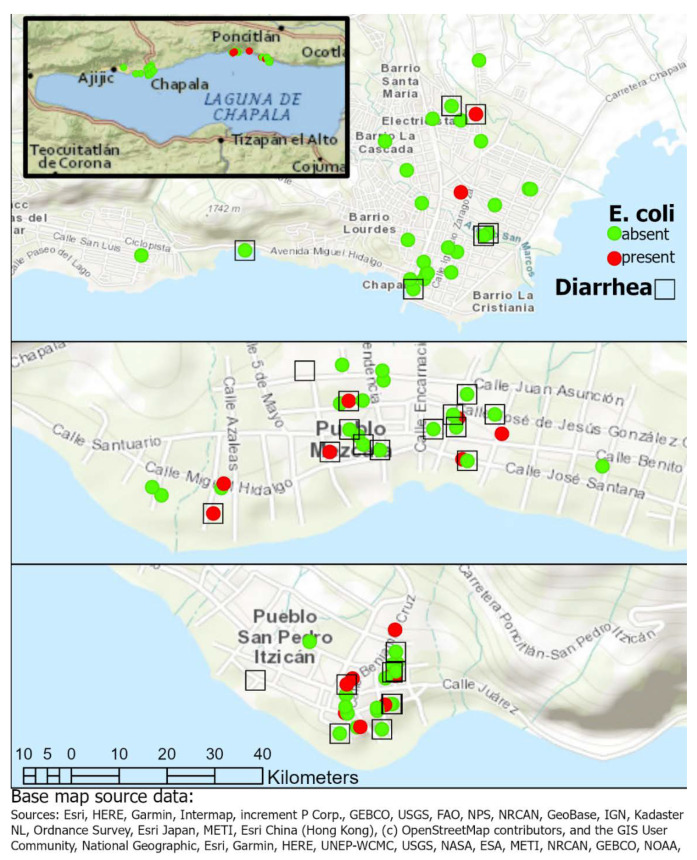
Presence of *E. coli* relative to diarrhea in Chapala (top), Mezcala (Middle) and San Pedro Itzicán (bottom). Inset shows the municipalities of Chapala and Poncitlán and relative locations of the three towns (left to right: Chapala, Mezcala, San Pedro Itzicán).

Figure 3 shows approximate locations of households with positive *E. coli* in the garrafones samples relative to self-reported diarrhea. Although Mezcala and San Pedro Itzicán had more positive *E. coli* than Chapala, within those two towns both the locations of households and the positive samples were randomly distributed according to the Gedis Ord Gi* statistic, *p* < 0.05.

## 4. Discussion

This mixed methods approach highlights drinking water quality as a serious issue in communities around Lake Chapala and highlights inadequate WASH as a key hazard potentially perpetuating high disease burden (both CKD and enteric disease) in the region. The occurrence of total coliform bacteria, *E. coli*, and arsenic in household water sources and drinking water garrafones strongly indicates that safely managed drinking water targets defined by the SDG 6 are not close to being achieved within our study communities.

The higher numbers of positive total coliform bacteria samples in Mezcala and San Pedro Itzicán were most likely due to purchase of improperly treated garrafones from local vendors as opposed to name-brand bottled water more widely available in Chapala. The fact that bacterial contaminants were found in garrafones in all three towns however, points to the need to further examine the point or points at which contamination occurs (during initial filling—i.e., from the source, during refilling, or within the home after the bottle has been opened). Although there appear to be differences between the number of *E. coli* positive samples between each town, we suspect that these differences are a function of the overall number of samples being positive for *E. coli* compared to total coliform bacteria (because not all Coliform Bacteria are *E. coli*). Survey respondents stated that they bought water from either of the two local small companies. The lack of clustering indicates a random spatial distribution of contaminated garrafones.

The findings reported here clearly support the belief of some community members that their drinking water is contaminated and require further investigation. Before this study, there were no data to support their concerns. Future work should be aimed at supporting routine monitoring and reporting of the microbial quality of both the primary source of water (bottled water), both at the well and within the bottling factory. Currently these small companies do not have the training nor facilities to do so. Under-resourced government agencies in these rural municipalities have not prioritized the oversight of bottled water.

High CKD and diarrheal disease prevalence among households when compared to regional and national estimates suggests that SDG 3 targets 3.3 and 3.4 are also far from being achieved. It is clear that additional financial, educational and healthcare resources are needed across Mezcala, Chapala, and San Pedro Itzicán to reduce mortality from non-communicable diseases through prevention and treatment as well as combat water-borne disease.

Household survey respondents in all three towns recognized the deteriorating water quality of the Lake Chapala basin, yet feel they do not have the financial resources nor appropriate knowledge to avoid exposing themselves or their families to dangerous chemical and microbial contaminants. In addition, the majority of respondents felt they are not getting enough support from the government for improving water quality and access. This awareness by study participants of the factors slowing progress toward safely managed drinking water and an overall healthier community is promising.

Although alternative drinking water sources are available in these three towns, our study results show that drinking water is not free from fecal and industrial contamination. Interventions aimed at public water systems would prevent these primarily low-income households from spending their limited funds on locally owned water purification companies operating under apparently non-existent quality control policies, management, oversight and transparency. It is imperative to focus the finite resources available in this region to address the issue of fecal and chemical contamination in drinking water. If this is done, it is possible that SDG target 6.1, and subsequently targets 6.3, 6B, 3.3 and 3.4 can be achieved.

The results reported herein are not without limitations, for example, the data collected on the outcomes of interest (CKD and diarrheal disease) were self-reported measures, and therefore a potential source of response bias. This is a concern related to the recall period used to assess diarrheal disease prevalence within a household. Research has shown that the longer the recall period, the greater the imprecision. However, our use of a two-week recall period attempted to address this shortcoming. As survey participants were responding on behalf of their entire household, respondents may have underestimated or overestimated diarrheal disease occurrence in their household [17]. There is no reason to assume that either misclassification occurred systematically and biased the results toward or away from the null, because there are factors that would favor the likelihood of either. Future studies may benefit by decreasing the recall period of diarrheal symptoms to a maximum of 7 days or obtaining stool samples to reduce subjectivity. Due to the time and resource constraints of this study, collecting stool samples was not possible. A hydrogeological study to determine whether arsenic contaminated water from industrial discharges flows to tributaries of Lake Chapala through the subsurface and into the wells could inform future studies of drinking water in these communities. While the findings are specific to the three towns included in the study, the mixed methods approach can be applied globally.

## 5. Conclusions

As outlined in this study, utilizing mixed methods can illuminate important gaps in the progress towards achieving the SDGs 3 and 6 by 2030. These gaps, which are highlighted in the Lake Chapala basin, include but are not limited to determining disease burden estimates, lack of access to not only “improved” but also safely managed drinking water free of chemical and microbial contamination. Participant perspectives added important depth to interpreting the quantitative data, as they included the lived experiences related to these public health issues and promote cultural sensitivity for more effective data collection (for example, precisely identifying the drinking water source when several options are available). Mixed methods studies, as described herein, can increase the community-level knowledge base, for example, the community members did not know that the water they were purchasing from local bottling companies was sometimes contaminated with *E. coli*. Armed with these data, they can be empowered to demand more accountability on the part of the companies and government agencies charged with monitoring water quality.

## Figures and Tables

**Table 1:** Specific targets and indicators of Sustainable Development Goal (SDG) 3 and 6 [5,8].

#	SDG	Target	Indicators
3	Ensure healthy lives and promote well-being for all at all ages	(3.3) By 2030, end the epidemics of AIDS, tuberculosis, malaria and neglected tropical diseases and combat hepatitis, water-borne diseases and other communicable diseases	(3.3.5) Number of people requiring interventions against neglected tropical diseases
		(3.4) By 2030, reduce by one third premature mortality from non-communicable diseases through prevention and treatment and promote mental health and well-being	(3.4.1) Mortality rate attributed to cardiovascular disease, cancer, diabetes or chronic respiratory disease
6	Ensure availability and sustainable management of water and sanitation for all	(6.1) By 2030, achieve universal and equitable access to safe and affordable drinking water for all	(6.1.1) Proportion of population using safely managed drinking water services
		(6.3) By 2030, improve water quality by reducing pollution, eliminating dumping and minimizing release of hazardous chemicals and materials, halving the proportion of untreated wastewater and substantially increasing recycling and safe reuse globally	(6.3.2) Proportion of bodies of water with good ambient water quality
		(6B) Support and strengthen the participation of local communities in improving water and sanitation management	(6.B.1) Proportion of local administrative units with established and operational policies and procedures for participation of local communities in water and sanitation management

**Table 2:** Sociodemographic characteristics of households.

Characteristic	N	(%)
Household Location	99	
Chapala	32	32.32
Mezcala	32	32.32
San Pedro Itzicán	35	35.35
Head Household Highest Education Level Reached	97	
Primary school	38	39.18
Secondary school	16	16.49
Above secondary	43	44.33
Head Household Female Occupation	89	
No work	61	68.54
Labor/construction	2	2.25
Agriculture	0	0.00
Other	26	29.21
Head Household Male Occupation	79	
No work	14	17.72
Labor/construction	26	32.91
Agriculture	21	26.58
Other	18	22.78
Household Income Avg. Pesos (monthly)	85	
MXN 0–MXN 5000	32	37.65
MXN 5000–MXN 10,000	34	40.00
MXN 10,000–MXN 15,000	9	10.59
>MXN 15,000	10	11.76
Household Size	99	
1–2	14	14.14
3–5	37	37.37
6–8	31	31.31
>8	17	17.17
Soda Servings per Day	79	
0	18	22.78
<1	13	16.46
1–2	40	50.63
3–5	8	10.13
Cooking Fuel	95	
Gas	68	71.58
Wood	27	28.42
Protein Servings per Day	88	
<2	51	57.95
>2	37	42.05

**Table 3:** Household primary water source and water use practice.

Survey Question	N	(%)
What is your household’s primary drinking water source?	97	
Garrafón (purchased 20 L)	96	98.97
Tap	1	1.03
What is your household’s primary cooking water source?	99	
Garrafón (purchased 20 L)	80	80.81
Tap	19	19.19
What is your household’s primary bathing water source?	99	
Tap	97	97.98
Private Well	1	1.01
Stream	1	1.01

## Supplementary Materials

The following are available online at https://www.mdpi.com/1660-4601/17/22/8328/s1, Table S1: Survey Data.
